# Supplementary material for: Single-domain antibody-based protein degrader for synucleinopathies
Source: Mol Neurodegener. 2024 May 31;19:44. doi: 10.1186/s13024-024-00730-y (PMC11140919; doi:10.1186/s13024-024-00730-y)

## Supplemental Figure 1: Synthesis of single-domain antibody (sdAb)-based protein degrader.

sdAb 2D8 was conjugated to thalidomide (an E3 ligase ligand) using three different lengths of linkers [–(PEG)n–, n = 2, 4, 6] that were attached to the lysine residues of the sdAb. Both unmodified sdAb 2D8 and the three modified sdAbs contained Myc and His tags at the N-terminus. Western blots show the detection of the sdAbs by anti-Myc antibody (A) and anti-His antibody (B).


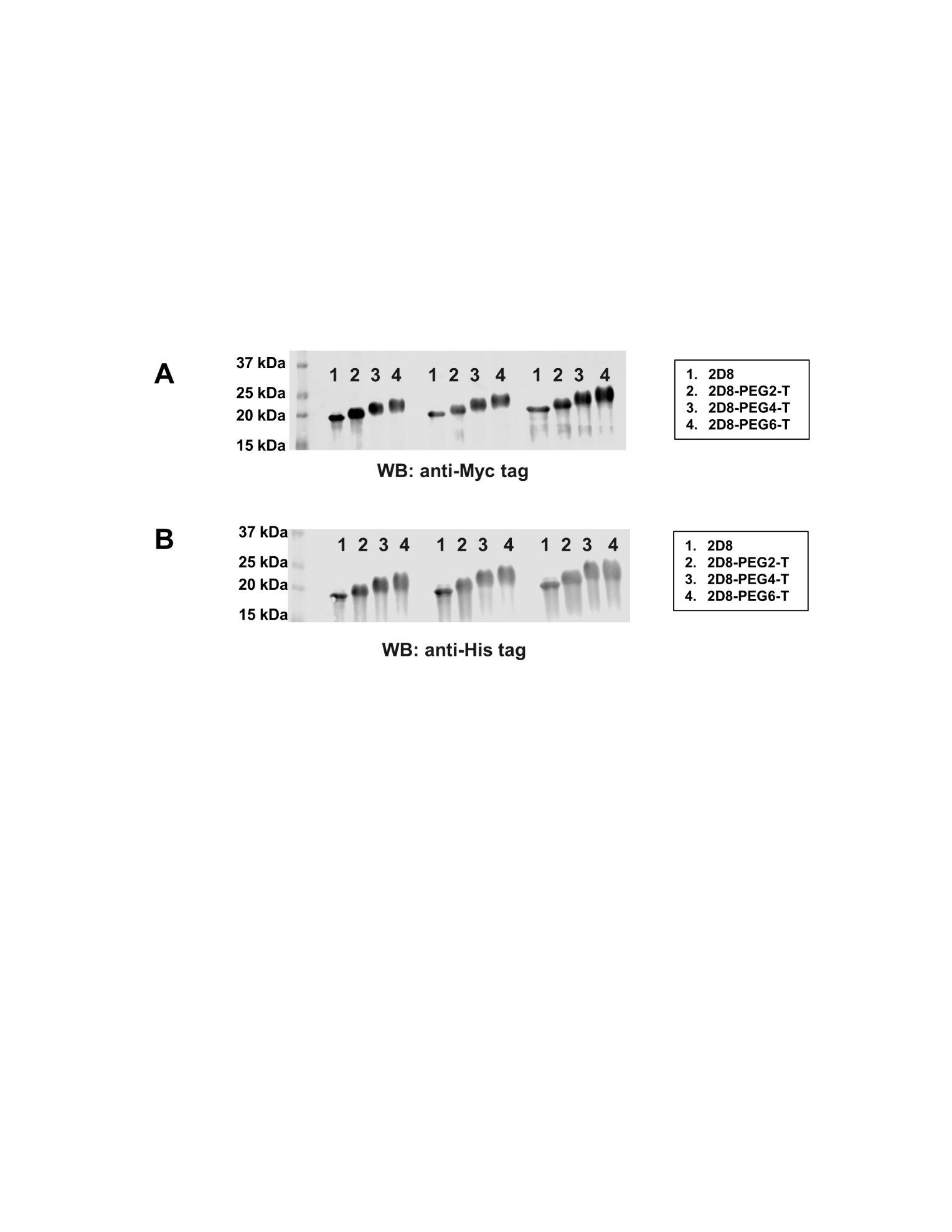


## Supplemental Figure 2: Assessment of toxicity of human Lewy body dementia (LBD) brain fractions.

(A) Schematic protocol illustrating the preparation of different fraction of α-syn from LBD brain. (B-C) Immunoblots illustrating GAPDH levels in treated cell lysate on different days (B). Toxicity of different α-syn fractions of LBD brain (C). Neuronal cultures were prepared from wild-type pups at day 0. Cells were incubated for 1, 2, 3, 5 and 7 days with 10 μg/ml of each of the six brain fractions. Toxicity was assessed by western blotting for GAPDH.

Day 1: A one-way ANOVA showed a significant overall treatment effect (p<0.0001). Cells treated with P1 fractions had significantly higher GAPDH levels compared to untreated control cells (p<0.0001).

Day 2: A one-way ANOVA showed a significant overall treatment effect (p<0.0001). Cells treated with either S2 (p=0.0375) and S3 (p<0.0001) fractions had significantly higher GAPDH levels compared to untreated control cells.

Day 3: A one-way ANOVA showed a significant overall treatment effect (p=0.0054). Cells treated with either S3 (p=0.0108) and P2 (p=0.0304) fractions had significantly higher GAPDH levels compared to untreated control cells.

Day 5: A one-way ANOVA showed a significant overall treatment effect (p<0.0001). The S1 fraction produced significant toxicity when compared to untreated control cells (p=0.0005). Cells treated with either the S2, S3, P2 and P3 fractions had significantly higher GAPDH levels compared to untreated control cells. (p=0.0169, p<0.0001, p<0.0001, p=0.036).

Day 7: A one-way ANOVA showed a significant overall treatment effect (p<0.0001). There was significant toxicity in cells treated with the S1 fraction (p=0.002). Cells incubated with S2 and P3 fractions showed higher levels of GAPDH relative to untreated controls (p<0.0001 for both).

**, ***, ****: p<0.01, 0.001, 0.0001 (Tukey’s post-hoc test).


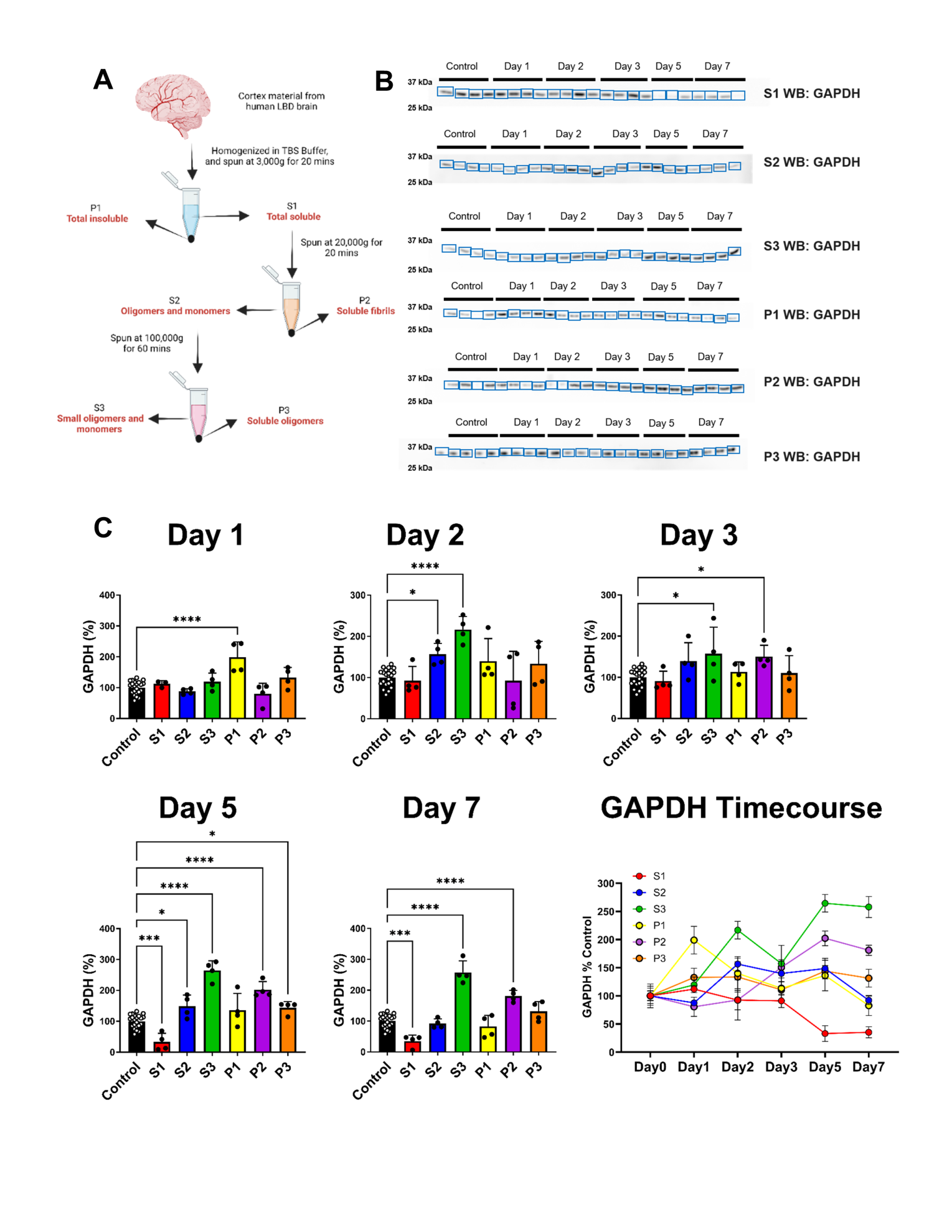


## Supplemental Figure 3: Immunoprecipitation (IP) depletion of α-syn from LBD S1-fraction and dot blot assay.

(A) Schematic protocol for IP and dot blot assays. The experimental details are described in the Materials and Methods section.

(B) Dot blot image and quantification. Positive control: rec α-syn; Negative control: bovine serum albumin (BSA). Dot blot analysis revealed significant differences in normalized signals between the groups (p<0.0001 for 2D8, 2D8-PEG2-T, 2D8-PEG4-T, and 2D8-PEG6-T; one-way ANOVA). Both 2D8 and modified 2D8 showed reduced binding after α-syn depletion, with binding decreasing by 90% (p<0.0001, 2D8), 92% (p<0.0001, 2D8-PEG2-T), 90% (p<0.0001, 2D8-PEG4-T), and 91% (p<0.0001, 2D8-PEG6-T) compared to S1, respectively. Notably, stronger binding of S1 (IP α-syn) was observed, with binding increasing by 86% (p<0.0001, 2D8), 88% (p<0.0001, 2D8-PEG2-T), 86% (p<0.0001, 2D8-PEG4-T), and 88% (p<0.0001, 2D8-PEG6-T) compared to S1 (IP α-syn flow through), respectively.


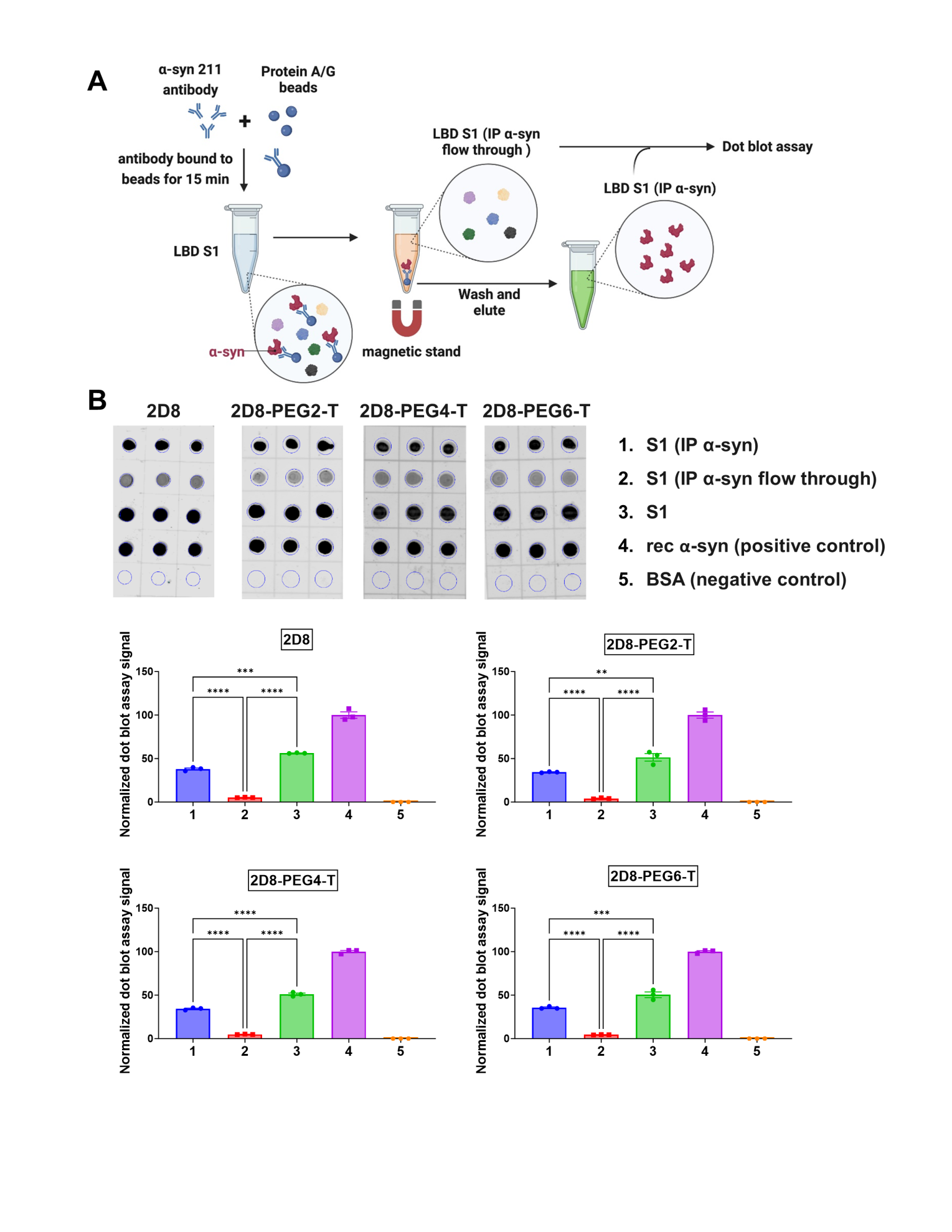


## Supplemental Figure 4: α-Syn seeding experiment.

Neuronal cultures were established from M83 mouse pups on day 0. Cells were treated with 10 µg/ml of LBD S1 fraction for 1, 2, 3, 5 and 7 days. The α-syn seeding capacity was evaluated using dot blot assays with conformational antibodies A11 (A) and OC (B). Quantitative analysis of the dot blot signals revealed significantly increased levels of α-syn aggregates in the presence of the LBD brain soluble S1 fraction (A11 antibody (C): p=0.0241 (day 3), p=0.0048 (day 5), p=0.0151 (day 7); OC antibody (D): p=0.0007 (day 3), p=0.0286 (day 5); unpaired t-test).


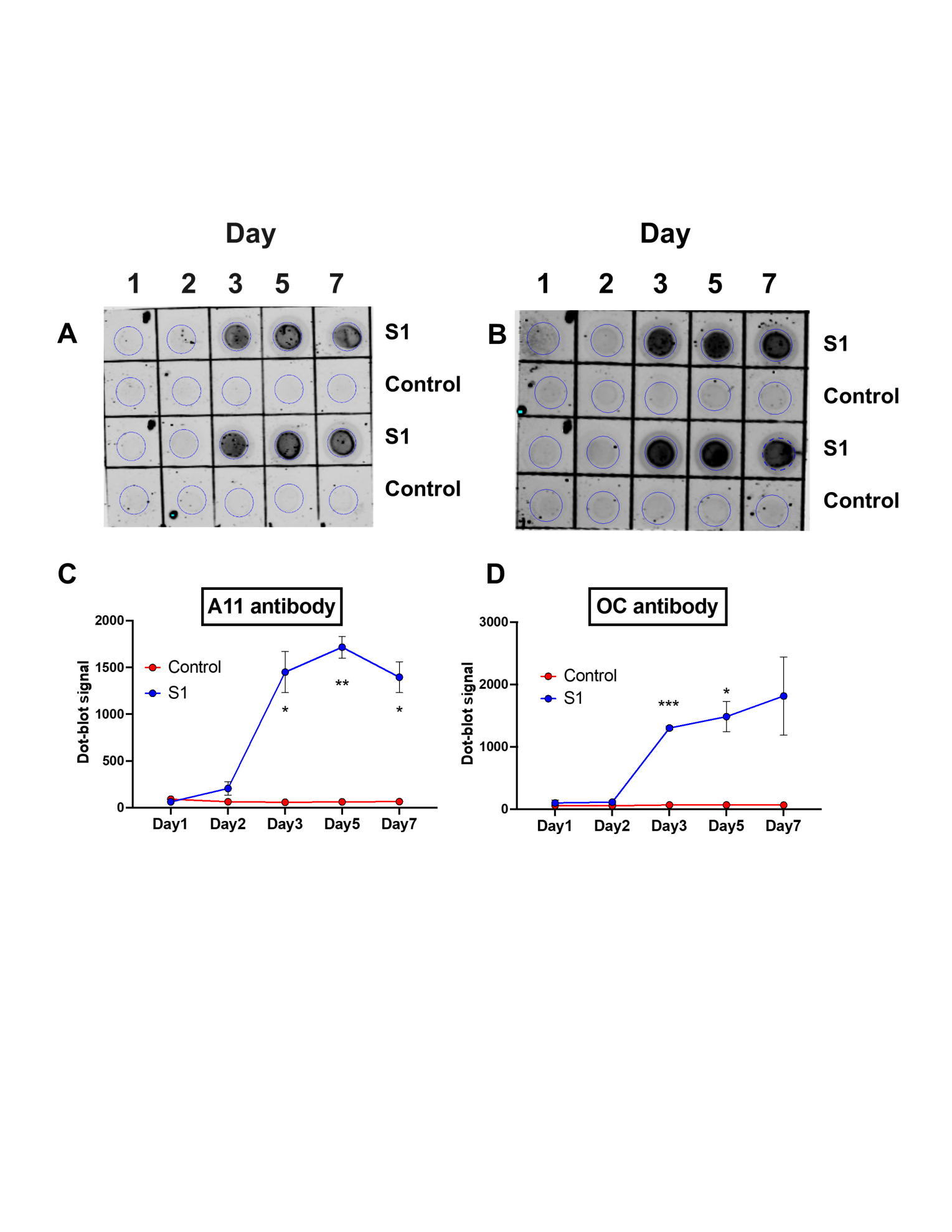


## Supplemental Figure 5: Complete western blots and bands quantified in Fig. 3.


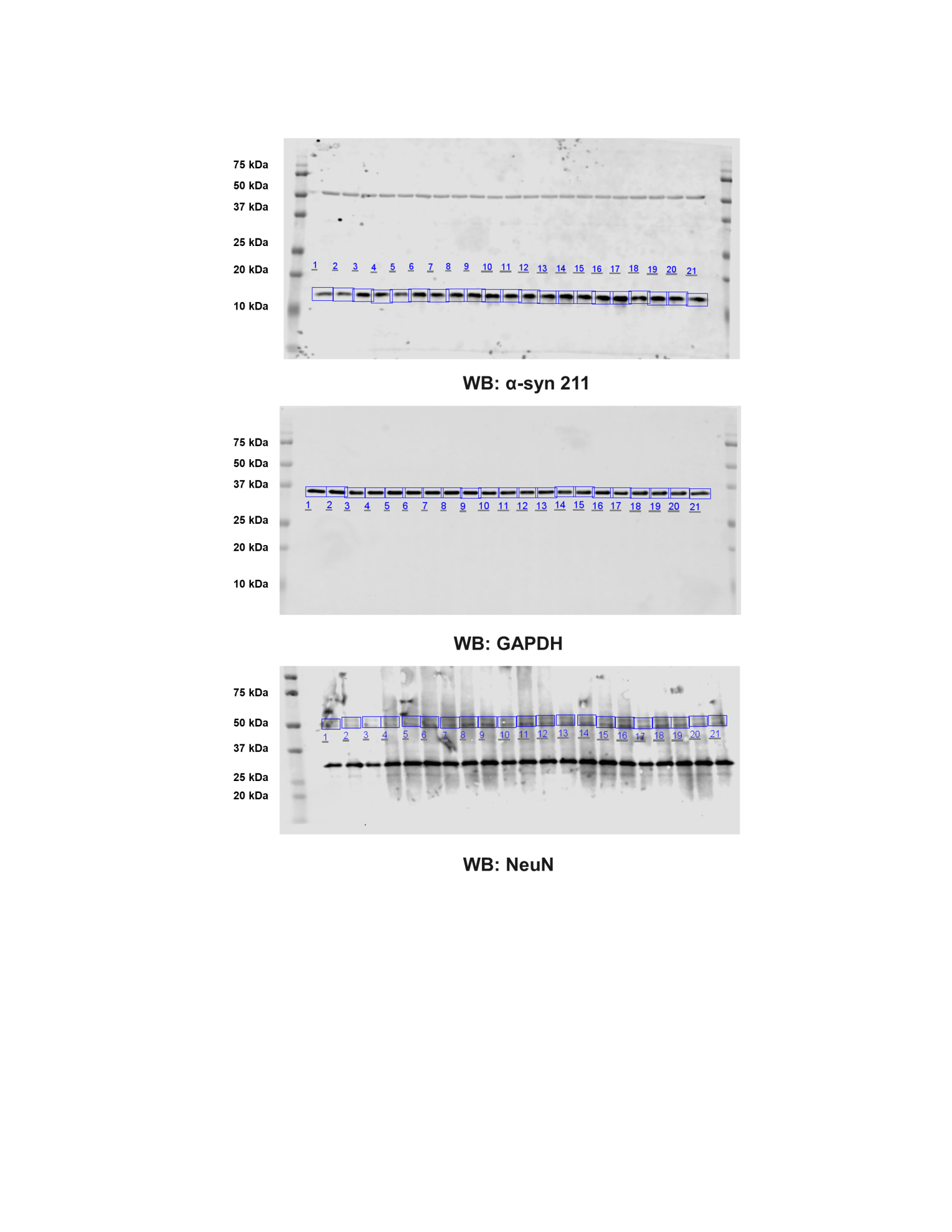


## Supplemental Figure 6: Complete western blots and bands quantified in Fig. 4.


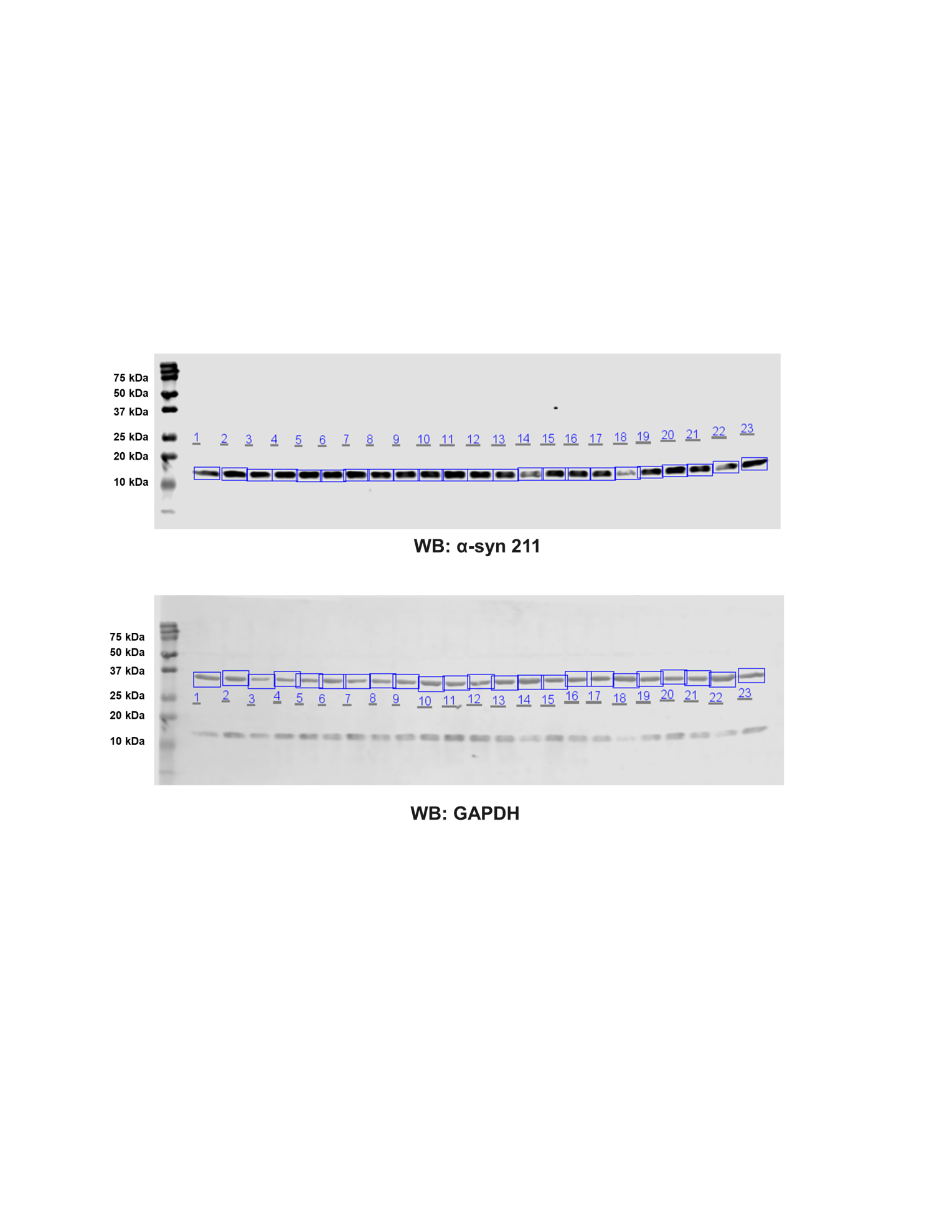


## Supplemental Figure 7: Complete western blots and bands quantified in Fig. 5.


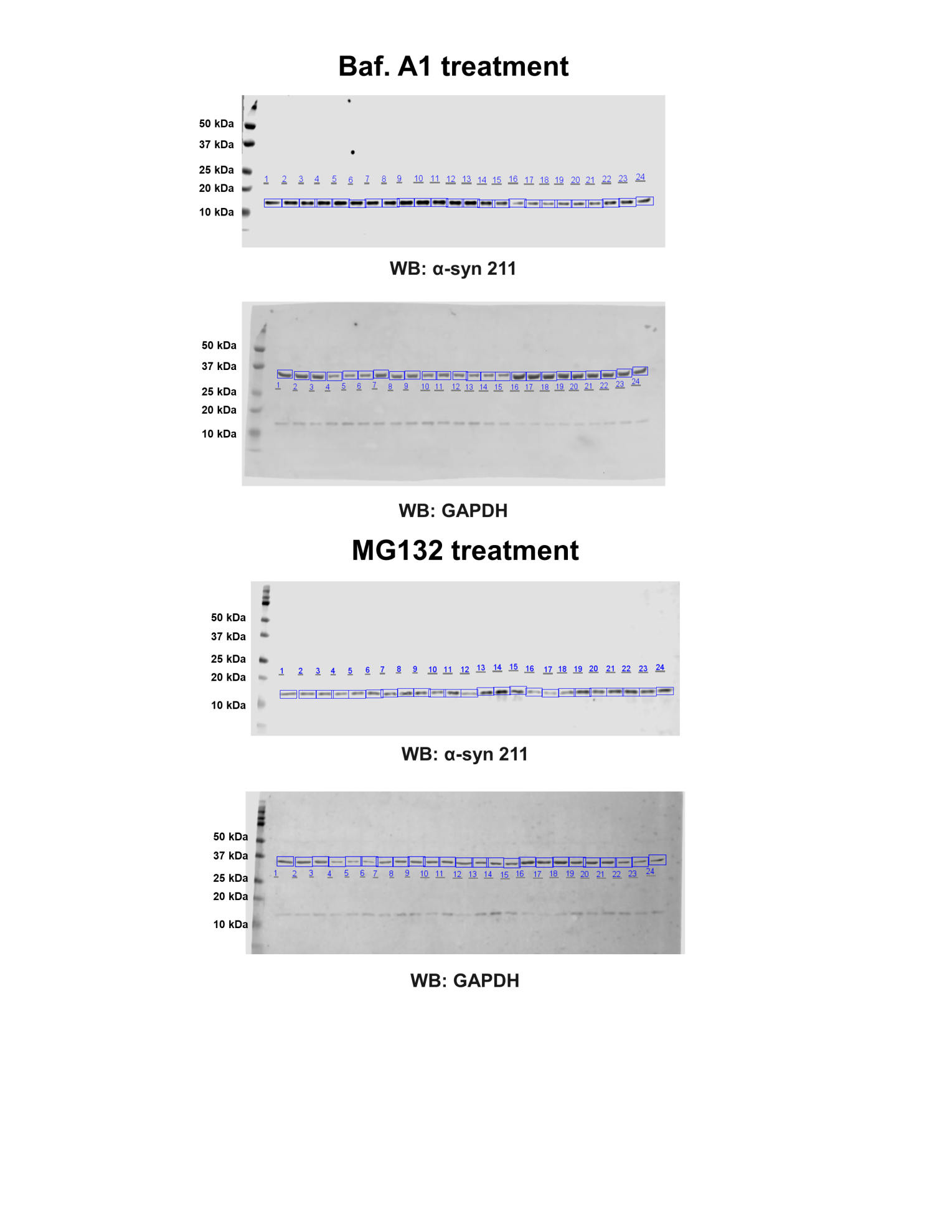


## Supplemental Figure 8: (A-B) Quantitation of the western bands presented in Figure 6 (C-D).

(A) Long conjugations to α-syn: Significant differences were observed between groups in long conjugations to α-syn (Figure 6C (i), one-way ANOVA, p=0.0017). The modified sdAb 2D8-PEG4-T with the proteasome inhibitor MG132 resulted in a 56% increase in long conjugations to α-syn compared to untreated sdAb 2D8 with MG132 (p=0.0245).

(B) Ubiquitin levels: Significant group differences were observed in ubiquitin levels (Figure 6D (i), one-way ANOVA, p<0.0001). The modified sdAb 2D8-PEG4-T significantly elevated ubiquitin levels by 53% (p=0.0064) and 43% (p=0.0208) compared to S1 alone and sdAb 2D8, respectively. Treatment with the proteasome inhibitor MG132 notably increased ubiquitin levels by 42% (p=0.027, sdAb 2D8 vs sdAb 2D8 + MG132) and 49% (p=0.0002, sdAb 2D8-PEG4-T vs sdAb 2D8-PEG4-T + MG132) for both unmodified and modified sdAb 2D8 groups. Furthermore, modified sdAb 2D8-PEG4-T combined with MG132 led to a 50% increase in ubiquitin levels compared to unmodified sdAb 2D8 with MG132 (p=0.0001).

(C-D) Complete Western Blots in Figure 6E.


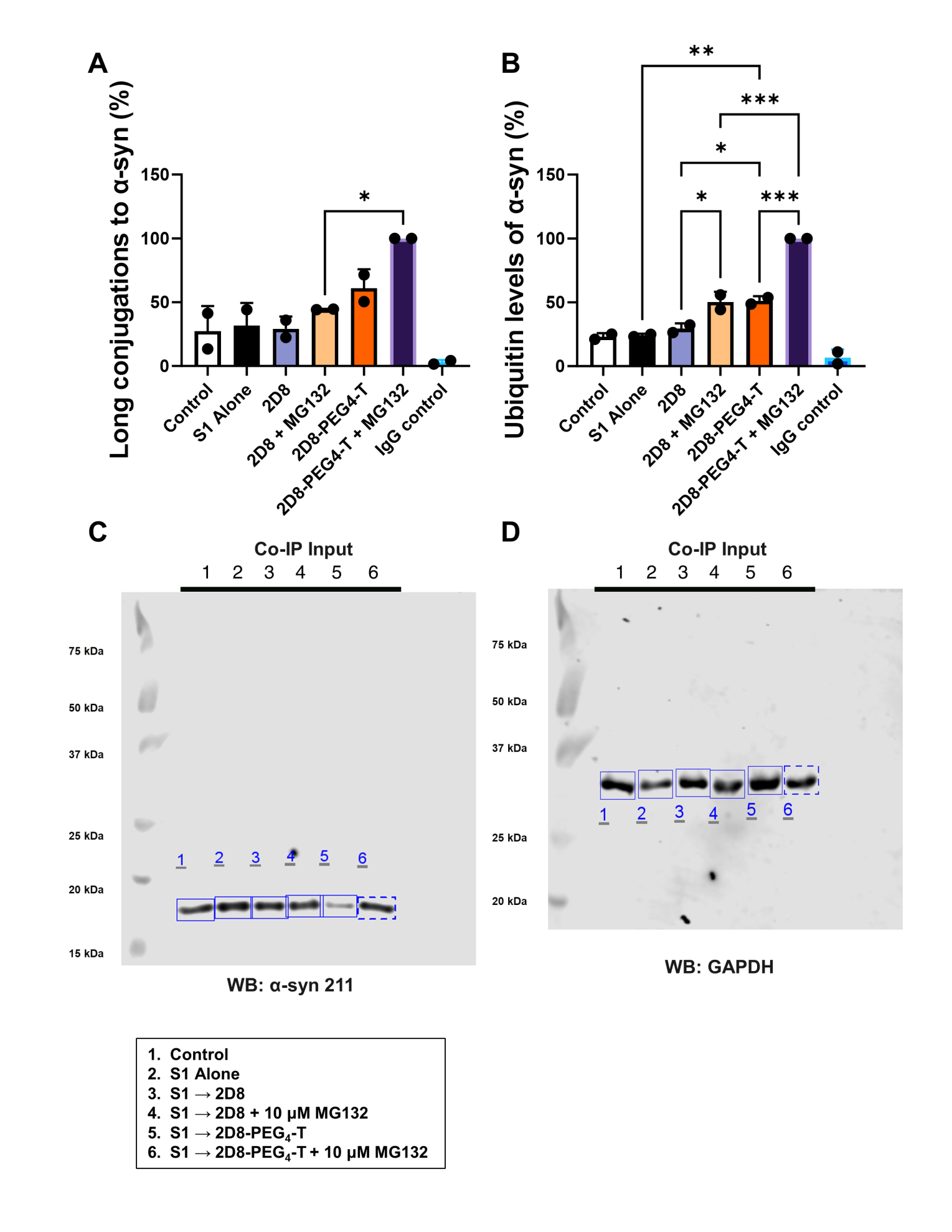


## Supplemental Figure 9: Brain-signal declines over days post single intravenous injection of near-infrared-labeled sdAb 2D8 (10 mg/kg).


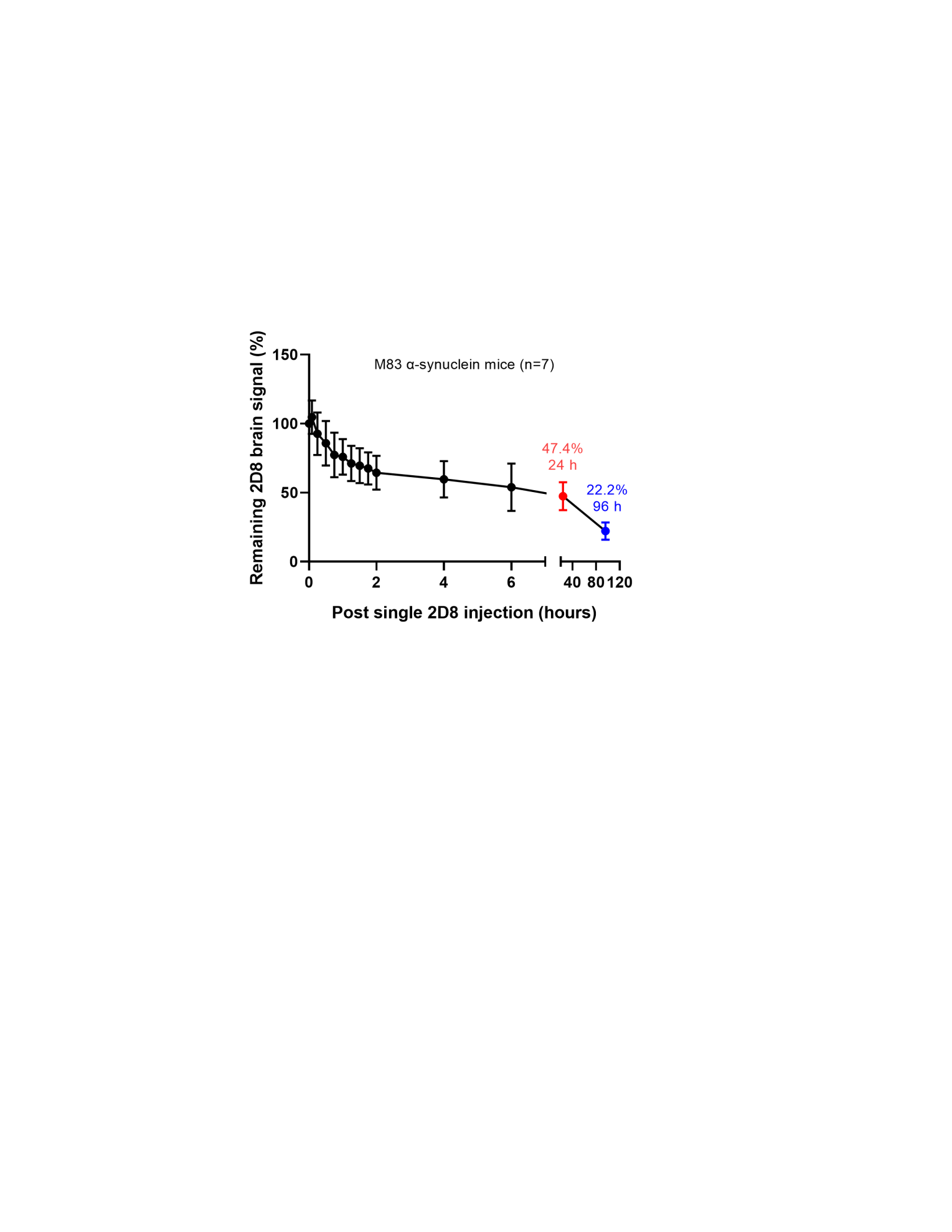


## Supplemental Figure 10: Complete western blots and bands quantified in Fig. 8-9.

The single band without a box in soluble α-syn 211 and GAPDH as well as insoluble α-syn 211 is an animal that was excluded from all analyses because of a very high IVIS signal throughout the body that appeared to be non-specific.


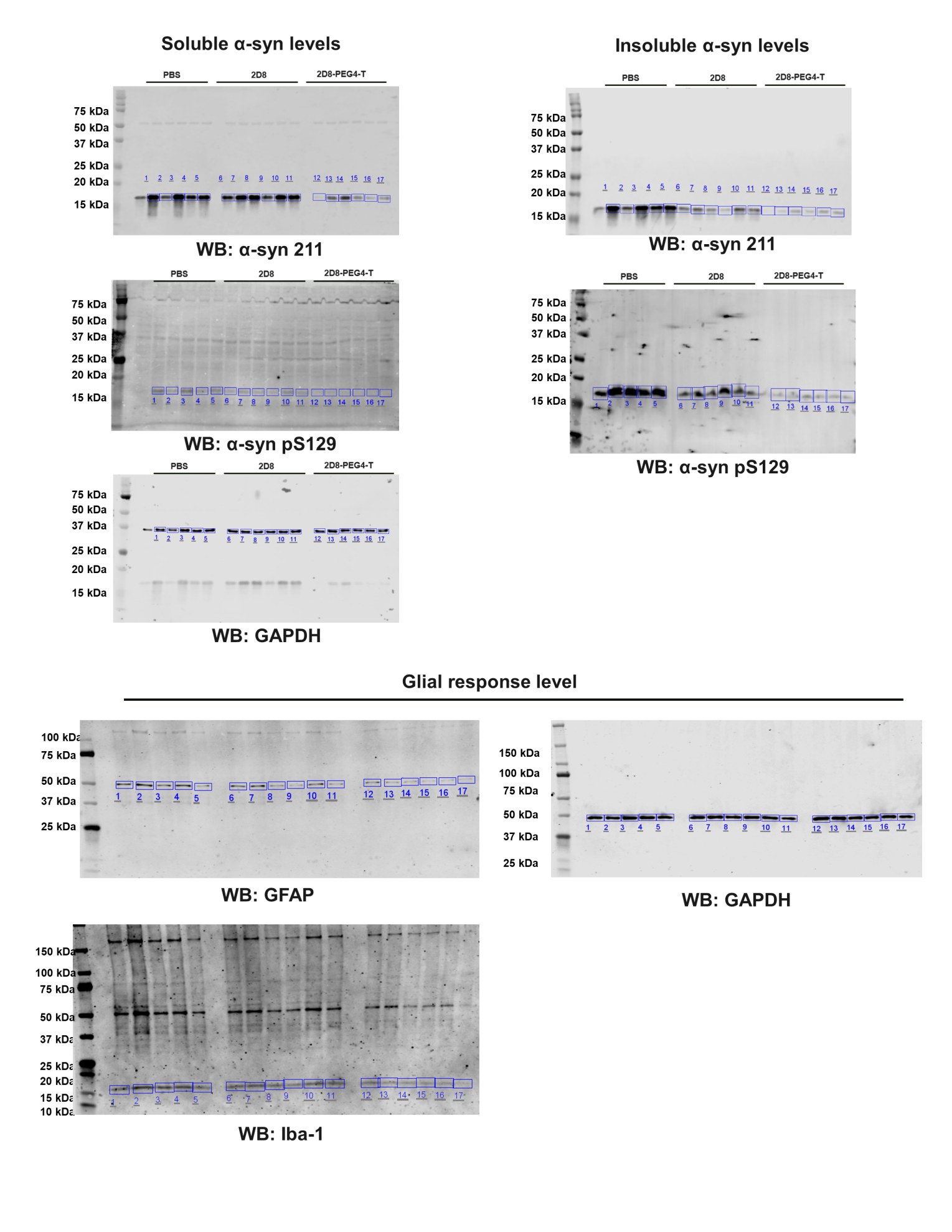

Supplement: Supplementary file 1 — Supplementary Material 1 [file 13024_2024_730_MOESM1_ESM.docx]
