# Supplementary material for: Single-domain antibody-based protein degrader for synucleinopathies
Source: Mol Neurodegener. 2024 May 31;19:44. doi: 10.1186/s13024-024-00730-y (PMC11140919; doi:10.1186/s13024-024-00730-y)
Supplement: Supplementary file 2 — Supplementary Material 2 [file 13024_2024_730_MOESM2_ESM.docx]

| **sdAb** | **Antigen** | **Phase** | **K_D_ (nM)** | **k_a_** | **k_d_** |
| --- | --- | --- | --- | --- | --- |
| 2D8 | rec α-syn | Solution phase | 41.2 ± 20.4 | 1.21 x 10^4^ | 4.84 x 10^-4^ |
| 2D8-PEG2-T | rec α-syn | Solution phase | 58.1 ± 26.9 | 1.50 x 10^4^ | 9.46 x 10^-4^ |
| 2D8-PEG4-T | rec α-syn | Solution phase | 66.7 ± 12.5 | 1.57 x 10^4^ | 1.07 x 10^-3^ |
| 2D8-PEG6-T | rec α-syn | Solution phase | 41.8 ± 20.5 | 5.90 x 10^3^ | 1.88 x 10^-4^ |
| 2D8 | LBD S1 | Solution phase | 84.1 ± 12.4 | 2.68 x 10^4^ | 2.34 x 10^-3^ |
| 2D8-PEG2-T | LBD S1 | Solution phase | 107.1 ± 41.4 | 8.42 x 10^3^ | 8.65 x 10^-4^ |
| 2D8-PEG4-T | LBD S1 | Solution phase | 85.9 ± 9.9 | 1.29 x 10^4^ | 1.10 x 10^-3^ |
| 2D8-PEG6-T | LBD S1 | Solution phase | 56.2 ± 21.1 | 2.31 x 10^4^ | 1.24 x 10^-3^ |

## **Supplemental** **Table 1. Binding results of unmodified and modified sdAb for different α-syn preparations.**

Binding, association, and disassociation constants of anti-α-syn sdAb 2D8, 2D8-PEG2-T, 2D8-PEG4-T and 2D8-PEG6-T, against rec α-syn and soluble fraction (S1) from LBD brain in solution phase measured by biolayer interferometry assay.

| **Treatment** | **Mouse #** | **Model** | **Age (months)** | **Sex** |
| --- | --- | --- | --- | --- |
| PBS | V34 | M83 (α-syn A53T) | 8 | M |
| PBS | V81 | M83 (α-syn A53T) | 8 | F |
| PBS | V62 | M83 (α-syn A53T) | 8 | M |
| PBS | V71 | M83 (α-syn A53T) | 8 | F |
| PBS | V28 | M83 (α-syn A53T) | 8 | M |
| 2D8 | V66 | M83 (α-syn A53T) | 8 | F |
| 2D8 | V79 | M83 (α-syn A53T) | 8 | M |
| 2D8 | V72 | M83 (α-syn A53T) | 8 | M |
| 2D8 | V82 | M83 (α-syn A53T) | 8 | F |
| 2D8 | V69 | M83 (α-syn A53T) | 8 | F |
| 2D8 | W8 | M83 (α-syn A53T) | 7 | M |
| 2D8-PEG4-T | V53 | M83 (α-syn A53T) | 8 | M |
| 2D8-PEG4-T | W24 | M83 (α-syn A53T) | 7 | F |
| 2D8-PEG4-T | W28 | M83 (α-syn A53T) | 7 | F |
| 2D8-PEG4-T | V31 | M83 (α-syn A53T) | 8 | M |
| 2D8-PEG4-T | W9 | M83 (α-syn A53T) | 7 | M |
| 2D8-PEG4-T | W23 | M83 (α-syn A53T) | 7 | F |

## **Supplemental Table 2: List of M83 synucleinopathy mice used for in vivo studies in Figures 7-9.**
